# Authors’ Response to Peer Reviews of “Social Media Polarization and Echo Chambers in the Context of COVID-19: Case Study”

**DOI:** 10.2196/32266

**Published:** 2021-08-05

**Authors:** Julie Jiang, Xiang Ren, Emilio Ferrara

**Affiliations:** 1 Information Sciences Institute University of Southern California Marina Del Rey, CA United States; 2 Department of Computer Science Viterbi School of Engineering University of Southern California Los Angeles, CA United States; 3 Annenberg School of Communication University of Southern California Los Angeles, CA United States

**Keywords:** social media, opinion, COVID-19, case study, polarization, communication, Twitter, echo chamber

*This is the authors’ response to peer-review reports for “Social Media Polarization and Echo Chambers in the Context of COVID-19: Case Study.”*

## Round 1 Review

### Reviewer L

#### General Comments

First, we would like to thank this reviewer [1] for their insightful comments on our paper [2]. Although endogeneity may be an issue of concern for these types of framings, our methodology builds on numerous studies (now cited in the revised paper) that—after controlling for many correlated variables—show how the emergence of online echo chambers is partly due to contagion dynamics, partly due to homophily, partly due to influence effects, and is not simply explained by one single mechanism (eg, political ideology alone). Nevertheless, our strategy has been proven effective to separate network structure from information spread dynamics. In the revised manuscript, we explained the various assumptions of the model, some potential limitations related to endogeneity, and referred to work illustrating the robustness of the adopted approach.

The reviewer is absolutely correct in that the real-world political ideology distribution may not match the one on Twitter. In fact, in the revised manuscript, we now refer to various studies that confirmed the same skewed online ideology distribution we observed in our study of Twitter. Since the data we observed is heavily left skewed, we used binning to facilitate comparison between left- and right-partisan users. This approach is consistent with prior work, which we now cite in the revised paper. We should note that as our study is restricted to Twitter, any insights we gleaned should only be assumed to be applicable to this platform—an important limitation that we now underscore in the revised manuscript, which, however, we do not think takes away from the importance of our work given the prominence of Twitter in political and public health discourse. The findings of how people share political information on Twitter may not necessarily generalize to other online platforms (or real-world offline networks). In the future, we will study the cross-platform dynamics of political information sharing. We clarified these limitations in the revised paper.

To clarify, we are not hypothesizing or postulating that COVID-19 (mis)information spreads differently from other information. We believe that studying the spread of (mis)information in the case of COVID-19 specifically is paramount due to the fact that it can have tangible effects on public health and how people behave in the offline world, with respect to health behaviors (eg, mask wearing, etc). We have illustrated some of these specific examples in the work we recently published (cited and further detailed in the revised manuscript). As for this specific paper, to avoid duplication, we limited the amount of discussion on specific content. Conversely, we wanted to concentrate specifically on the interplay between political ideology and COVID-19 online discourse to characterize how pre-existing polarization due to political divide may further exacerbate the spread of misinformation or potentially alter the dynamics of (factual and/or incorrect) information in the presence of echo chambers. To our knowledge, our study is the first to characterize this interplay and its effect on COVID-19 online discourse.

### Reviewer R

#### General Comments

We would like to thank this reviewer [3] for their feedback.

The motivation of the paper is to understand the role social media polarization plays in contributing (mis)information spread regarding COVID-19. This is of particular importance currently as inaccurate information may undermine public health efforts. Since prior works show that attitude toward COVID-19 is linked to political ideology, understanding the extent of polarization will be helpful for relaying information and debunking misinformation. In the revised manuscript, we added to the Introduction to strengthen our motivation for the paper, as well as to the Discussion for an in-depth discussion of the implications of our work.

We added more detailed explanations for all the models mentioned in the paper, including word embeddings, transformers, S-BERT, and network embeddings in the Methods section.

#### Specific Comments

##### Major Comments

1. Thank you for this comment. In our revised manuscript, we clarified the research questions to better reflect their relevance to COVID-19.
2. Thank you for this comment. In “Related Work,” we have added explanations of word embeddings, transformers, and network embeddings so that readers can have a high-level understanding of these models.
3. Thank you for this comment. In our revised manuscript, we have added more layman explanations of each model when we introduce them in the Methods section. We believe this helps give readers a more intuitive understanding of word embeddings, transformers, etc.
4. Thank you for this comment. We removed the most bot-like users as is customarily done when dealing with potentially bot-infused data. If bots infiltrated users of different partisanship equally, then we expect to find a similar distribution of bot scores across all users. Since this is not what Figure 2B shows, it suggests there may be more right-leaning bots. In our revised manuscript, we clarified what we expect to find to highlight what we observed in terms of bot score distributions.
5. We thank the reviewer for this insight. In our paper, we use the term “partisan users” to refer to users who are strong supporters of a party, which could be very left or very right. As such, this is corroborated precisely by the U-shaped distributions in Figure 3B, C, and D. Figure 3A and E only shows that left-leaning users are influential, which could be attributed to Twitter’s left bias as a platform (giving more verified status to left-leaning users) and the larger left-leaning user base. In our revised manuscript, we added a suggestion that the phenomenon could be attributed to the large left-leaning user base.
6. Thank you!
7. Thank you!
8. We agree with the reviewer’s comment. We have added a paragraph on the implications of our work for health and wellness.

##### Minor Comments

1. We defined “AUC” along with a short explanation of why it was chosen (over accuracy, etc).
2. “NLP” has been replaced with “natural language processing.”
3. The reviewer is right; this mistake in the caption has been corrected.
4. Thank you. You can find out more about this from:
  Garimella K, De Francisci Morales G, Gionis A, Mathioudakis M. 2017. Quantifying controversy on social media. ACM Trans Soc Comput, 1 (1) Article 1. DOI: 10.1145/3140565
